# Network-Aware Control Barrier Functions for Resilient Microgrids Under Stealthy Drift Attacks

**DOI:** 10.3390/s26144329

**Published:** 2026-07-08

**Authors:** Mordecai Opoku Ohemeng, Frederick T. Sheldon

**Affiliations:** Department of Computer Science, University of Idaho, Moscow, ID 83843, USA; sheldon@uidaho.edu

**Keywords:** microgrids, Lyapunov–Krasovskii functional, control barrier functions, cyber–physical security, stealthy drift attacks, input-to-state stability

## Abstract

Inverter-dominated microgrids are highly vulnerable to stealthy cyber–physical drift attacks, low-amplitude, slowly varying perturbations that bypass conventional statistical filters to induce voltage degradation and delayed collapse. This paper introduces a resilient, delay-aware supervisory control architecture that acts as an online safety shield at the actuator interface. By jointly modeling nonlinear power-flow interactions and directional communication topologies, we construct physics-informed Control Barrier Functions (CBFs), embedding structural electrical invariants derived from the nodal admittance matrix $Y_{bus}$. The supervisor directly incorporates heterogeneous, time-varying network delays into its safety constraints and utilizes a threat-adaptive modulation loop driven by spatio-temporal residuals to dynamically scale intervention aggressiveness. Using a Lyapunov–Krasovskii functional, we prove that the closed-loop tracking error is Input-to-State Stable (ISS) under bounded drift and worst-case latencies. High-fidelity simulations on an IEEE 14-bus test feeder demonstrate that the supervisor consistently enforces non-negative safety margins and reduces time-integrated voltage violations. Under coordinated sub-threshold attacks designed to exploit network jitter, the architecture bounds trajectories to physically consistent manifolds and prevents voltage collapse, establishing a scalable cross-layer safety framework for resilient distribution systems.

## 1. Introduction

Modern distribution systems are undergoing a rapid transformation, which is driven by the proliferation of distributed energy resources (DERs), electrification of end-use loads, and the widespread deployment of inverter-based resources (IBRs) [1,2]. These IBRs rely on fast-acting power electronic interfaces whose performance is coordinated through distributed or hierarchical secondary control loops. In practice, these loops depend on continuous cyber–physical interactions, frequency and voltage measurements, power setpoints, and synchronization signals that are packetized and exchanged across heterogeneous communication networks [3,4]. While this digitalization enables autonomous voltage regulation, economic dispatch, and improved situational awareness, it also exposes the physical grid to cyber-layer vulnerabilities that can directly compromise operational safety.

A key challenge is that the cyber and physical layers evolve on comparable timescales. Even small perturbations in communication channels, such as delayed, corrupted, or biased control packets, can propagate through the nonlinear inverter dynamics and destabilize the microgrid. Recent studies [5] have shown that adversaries increasingly exploit this by injecting stealthy drift perturbations (low-amplitude, slowly varying biases), which mimic natural load changes or benign network fluctuations. These attacks evade conventional intrusion detection systems (IDSs), statistical bad-data detectors, and threshold-based anomaly filters, and gradually steer the system toward voltage collapse or frequency instability.

### 1.1. Literature Review and Related Work

The security of decentralized cyber–physical systems (CPSs) and inverter-dominated microgrids against sophisticated network perturbations has generated several distinct research fields. In the realm of cyber–physical anomaly detection and mitigation, the work by [5] introduced a physics-constrained optimization framework for mapping stealthy drift perturbations, proving that intelligent adversaries can exploit the algebraic nullspace of network topology matrices to remain undetected while actively degrading system stability margins. This foundational threat vector is echoed by [6], who demonstrated that secondary control loops are structurally vulnerable to coordinated cyber-attacks that subtly warp physical voltage references without triggering conventional residual thresholds.

To bridge this cyber–physical divide, researchers have increasingly turned to advanced data-driven, localized, and decentralized validation paradigms. For instance, blockchain-augmented architectures have been introduced by [7] to mitigate False Data Injection (FDI) attacks and enhance cross-layer infrastructure resilience. Simultaneously, intelligent sensing frameworks utilizing Multi-Head Self-Attention Long Short-Term Memory (MHSA-LSTM) networks have shown remarkable efficacy in isolating localized data-driven anomalies, such as ransomware injections in critical telemetry layers [8].

Furthermore, traditional protection schemes often assume observable, high-frequency signatures. For instance, while [9] developed effective oscillator-based detection architectures for distributed secondary control, their reliance on clear oscillatory footprints leaves them blind to slow, sub-threshold geometric drift. This vulnerability is expanded at the system layer by [10], who highlighted the compounding dangers of multi-link coordinated attacks that target communication topology asymmetries, a challenge further compounded by the reality that traditional information technology perimeter barriers cannot defend real-time operational states governed by nonlinear physical dynamics [11].

#### 1.1.1. Evolution of Control Barrier Functions (CBFs)

To translate anomaly detection into proactive, mathematically certified safety enforcement, CBFs have emerged as a dominant safety-filtering methodology. Standard Ames-type CBFs enforce the forward invariance of a safe operating set C by restricting control laws via linear inequality constraints evaluated in an optimization loop [12], which has seen tailored deployment in decentralized voltage control for AC microgrids [13]. Despite their elegance, standard CBF formulations face significant practical deployment challenges. A comprehensive review by [14] outlines key theoretical gaps in conventional safe synthesis, including the inability to natively guarantee safety under strict input constraints, high-degree relative dynamics, and non-minimum phase system topologies. To bridge performance and safety, data-driven alternatives have also been explored. Notably, recent advances in safe off-policy deep reinforcement learning for Volt-VAR control [15] focus heavily on optimizing operational performance under dynamic grid conditions. However, these reinforcement learning paradigms rely on statistical reward functions rather than deterministic safety certificates, leaving them inherently brittle and vulnerable to malicious, sub-threshold cyber–physical perturbations.

To cope with complex plant models, the recent literature has shifted toward decoupled architectures. The work in [16] proposed evaluating safety-critical constraints via reduced-order models (ROMs), utilizing a performance–safety trade-off layer to manage discrepancies between simplified control models and physical system states. To address the conservative nature of standard barrier boundaries, the work by [17] introduced optimizable CBFs that dynamically adapt boundary constraints to maximize feasibility margins and expand behavioral diversity while guaranteeing absolute safety. Furthermore, extending safety filters to non-deterministic domains has led to the formalization of stochastic CBFs by [18], which mitigate risk in the presence of continuous Brownian noise or stochastic disturbance vectors.

#### 1.1.2. The Challenge of Time Delays and Input Constraints

A critical limitation of the aforementioned literature is the assumption of instantaneous, delay-free state feedback and control execution. In practical networked microgrids, transport lag, variable routing queues, and stealthy jitter introduce severe phase margin erosion. To handle these multi-variable stresses, early control frameworks explored the integration of Control Lyapunov Functions (CLFs) and CBFs. The work by [19] presented a combined Control Lyapunov–Barrier Function (CLBF) framework capable of ensuring asymptotic stabilization while maintaining geometric set safety under bounded state perturbations.

However, when communication paths introduce uncompensated latencies, the safety filter itself can become a source of closed-loop instability. Addressing this, the work in [20] developed a predictor-based CBF framework specifically for linear systems with input delays, showing that forward state prediction is required to maintain safety boundaries when a pure time delay τ shifts the control execution. To circumvent the need for precise predictive models, the work in [21] introduced Adaptive Control Barrier Functions (aCBFs) that dynamically update system parameter estimates in real time, absorbing external plant variations. Yet, these adaptive frameworks do not integrate parameter-dependent damping loops capable of counteracting the specific right-half plane (RHP) zeros introduced by transcendental network delays.

#### 1.1.3. Research Gaps and Core Contributions

By cross-examining current state-of-the-art methods, three distinct structural gaps are identified in the literature:

1. CBFs in the current literature assume perfect, delay-free feedback. They do not incorporate heterogeneous, non-minimum phase communication latencies into their safety guarantees, causing the barrier filters to become closed-loop unstable under practical latencies.
2. Existing advanced sensing and machine learning frameworks excel at isolating attacks or sub-threshold drift but operate strictly as passive detection systems, lacking the ability to translate detection parameters into real-time corrective control inputs.
3. Conventional delay-aware methods rely heavily on predictive states or assume linear, perturbation-free plants. No unified framework seamlessly balances dynamic load variance, stealthy cyber-drift, and latency metrics within a single parameter-dependent, active supervisor layer.

To explicitly demonstrate how this manuscript bridges these gaps, Table 1 summarizes the limitations of previous prominent architectures and contrasts them against the proposed framework.

### 1.2. Proposed Work and Key Contributions

To address these gaps, this paper introduces a resilient, delay-aware supervisory control architecture that acts as an online safety shield for inverter-dominated microgrids. Rather than relying on standard delay-oblivious safety filters found in the conventional literature, which fail under transport lag, the supervisor is deployed directly at the actuator interface. It intercepts potentially compromised control commands and filters them through a real-time QP constrained by adaptive, physics-informed CBFs. The key contributions of this work are summarized as follows:

1. We develop a framework that captures the structural asymmetries between physical power-flow couplings and directional cyber-communication links, enabling a representation of cyber–physical interactions under attack.
2. We construct a class of CBFs that embed algebraic network invariants derived from $\ker(Y_{bus})$ and explicitly incorporate heterogeneous, time-varying communication delays into the safety constraints, directly counteracting the RHP zeros introduced by transport latency.
3. We introduce a dynamic modulation loop that couples the CBF safety margin with spatio-temporal residuals. This mechanism tightens or relaxes safety boundaries based on real-time threat levels, enabling minimally invasive operation under nominal conditions and aggressive intervention under attack.
4. Using a Lyapunov–Krasovskii functional, we prove that the tracking error is Input-to-State Stable (ISS) with respect to both bounded drift perturbations and worst-case network delays, ensuring robust stability and formal safety guarantees of the closed-loop system.

Overall, this work provides an architecture capable of mitigating stealthy drift attacks in inverter-dominated microgrids while preserving nominal performance and ensuring real-time operational safety.

## 2. Cyber–Physical System (CPS) Modeling and Threat Framework

This section formalizes the cyber–physical structure of the networked microgrid and introduces the adversarial drift model used throughout the analysis. The framework integrates a physics-based representation of inverter dynamics and network flow constraints, a communication-layer model capturing routing delays and packet corruption, and a stealthy, sub-threshold drift attack designed to evade conventional anomaly detectors.

### 2.1. Dual-Graph Cyber–Physical Architecture

The microgrid is modeled as a dual-graph system $\left(G_{ph},G_{cy}\right)$ that captures the interaction between electrical power flows and networked control signals.

#### Physical Layer

The physical distribution network is represented by an undirected graph $G_{ph}=\left(V_{ph},E_{ph}\right)$, where $V_{ph}=\left\{1,2,\ldots ,N\right\}$ denotes the electrical buses and $E_{ph}$ denotes the transmission lines. Each IBR at bus *i* follows nonlinear control-affine dynamics:

(1) $${\dot{x}}_{i}\left(t\right)=f_{i}\left(x_{i}\left(t\right)\right)+g_{i}\left(x_{i}\left(t\right)\right)u_{i}^{net}\left(t\right),$$

where $x_{i}\left(t\right)={\left[\Delta \omega_{i}\left(t\right),\;\theta_{i}\left(t\right),\;V_{i}\left(t\right)\right]}^{T}$ contains the local frequency deviation (ω), phase angle (θ), and voltage magnitude (*V*). The networked control input $u_{i}^{net}\left(t\right)={\left[P_{i,ref},\;Q_{i,ref}\right]}^{T}$ represents the active(*P*) and reactive (*Q*) power setpoints delivered to the inverter interface.

Stacking all local states yields the aggregate nonlinear microgrid model:

(2) $$\dot{x}\left(t\right)=f\left(x\left(t\right)\right)+g\left(x\left(t\right)\right)u^{net}\left(t\right),$$

which forms the basis for the safety analysis and CBF supervisor design.

### 2.2. Network Physics Invariants

The physical layer is constrained by Kirchhoff’s laws and the structural properties of the network admittance matrix. Let $Y_{bus}\in C^{N\times N}$ denote the nodal admittance matrix. The complex voltage vector V and current injection vector I must satisfy:

(3) $$I=Y_{bus}V.$$

Under nominal operation, the steady-state trajectories of the microgrid lie on a physically consistent manifold:

(4) $$x\left(t\right)\in M\subseteq \ker\left(Y_{bus}\right),$$

where M encodes feasible voltage–current relationships. Departures from M indicate either abnormal loading, inverter saturation, or cyber-induced corruption of control signals. This plays a key role in the construction of the physics-informed barrier function used by the supervisor.

### 2.3. Networked Communication Layer and Delay Model

The cyber layer is modeled as a directed communication graph $G_{cy}=\left(V_{cy},E_{cy}\right)$, where each edge $\left(j,i\right)\in E_{cy}$ represents a unidirectional communication link carrying control packets from node *j* to node *i*. Each packet experiences a time-varying delay $\tau_{ji}\left(t\right)$ due to routing, buffering, and network congestion:

(5) $$u_{i}^{net}\left(t\right)=u_{i,nom}\left(t-\tau_{ji}\left(t\right)\right).$$

We assume:

(6) $$0\leq \tau_{ji}\left(t\right)\leq \tau_{\max},\;{\dot{\tau }}_{ji}\left(t\right)<1,$$

ensuring that the delay does not grow faster than real time. This model captures realistic communication behavior in distribution systems using mixed media (fiber, PLC, wireless), where delays are bounded but non-negligible.

### 2.4. Stealthy Drift Threat Model

We consider an intelligent adversary capable of compromising a subset of communication links $E_{cy}^{*}\subset E_{cy}$ within the networked microgrid. Rather than injecting large, abrupt false-data packets that would be easily detected, the attacker introduces a low-amplitude, slowly varying drift signal designed to mimic natural load fluctuations or benign network jitter. The corrupted control packet received at inverter *i* is modeled as

(7) $$u_{i}^{net}\left(t\right)=u_{i,nom}\left(t-\tau_{ji}\left(t\right)\right)+B_{c,i}d_{i}\left(t\right),$$

where $u_{i,nom}\left(t-\tau_{ji}\left(t\right)\right)$ is the delayed nominal reference generated by the secondary controller, and the term $B_{c,i}d_{i}\left(t\right)$ represents the adversarial perturbation.

The vector $d_{i}\left(t\right)\in R^{m_{i}}$ denotes the malicious drift signal injected into the communication channel feeding inverter *i*. This drift is assumed to be bounded and slowly varying:

(8) $$∥d_{i}{\left(t\right)∥}_{\infty }\leq \delta ,\;{\dot{d}}_{i}\left(t\right)\approx 0,$$

ensuring that the attack remains sub-threshold and evades conventional statistical anomaly detectors. The matrix $B_{c,i}\in R^{2\times m_{i}}$ is the cyber-attack input mapping matrix that determines how the drift enters the inverter interface. If the attacker corrupts both the active and reactive power references, then

(9) $$B_{c,i}=\left[\begin{array}{cc} 1 & 0\\ 0 & 1 \end{array}\right],$$

whereas corruption of only the active-power channel would correspond to

(10) $$B_{c,i}=\left[\begin{array}{c} 1\\ 0 \end{array}\right].$$

Stacking all compromised communication channels yields the following global representation of the attacked microgrid dynamics.

Let $d\left(t\right)={\left[d_{1}^{T}\left(t\right),\ldots ,d_{N}^{T}\left(t\right)\right]}^{T}\in R^{m}$ denote the aggregated adversarial drift vector, where $d_{i}\left(t\right)\in R^{m_{i}}$ is the local drift injected into the communication link feeding inverter *i*. The corresponding cyber-attack input mapping matrix is constructed as the block-diagonal operator

(11) $$B_{c}=\mathrm{blkdiag}\;\left(B_{c,1},B_{c,2},\ldots ,B_{c,N}\right)\in R^{2N\times m},$$

where each $B_{c,i}\in R^{2\times m_{i}}$ determines which components of the active/reactive power reference vector $u_{i}^{net}\left(t\right)$ are susceptible to corruption. The block-diagonal structure ensures that adversarial perturbations injected at different buses remain localized to their respective control channels unless explicitly coordinated by the attacker.

Using this notation, the continuous-time evolution of the microgrid under stealthy drift perturbations is expressed compactly as

(12) $$\dot{x}\left(t\right)=f\left(x\left(t\right)\right)+g\left(x\left(t\right)\right)\;u_{nom}\left(t-\tau \left(t\right)\right)+B_{c}\;d\left(t\right),$$

where $u_{nom}\left(t-\tau \left(t\right)\right)$ denotes the delayed nominal control packet received from the secondary controller and τ(t) represents the vector of heterogeneous, time-varying communication delays across the cyber layer. This captures how low-amplitude, slowly varying drift signals propagate through the networked control architecture and gradually steer the physical state x(t) away from the safe operating manifold defined by the microgrid’s electrical invariants.

#### Sub-Threshold Evasion

The adversary shapes d(t) to remain undetected by the baseline model, which computes a spatio-temporal anomaly score R. The attacker ensures:

(13) $$R\;\left(\left\{x\left(t-\tau_{ji}\right)\right\},\;u^{net}\left(t\right)\right)\leq \epsilon ,$$

where ϵ is the detection threshold. By keeping R sub-threshold, the attacker gradually steers the system away from the safe manifold M toward voltage or thermal collapse without triggering alarms.

This threat model captures the most dangerous class of cyber–physical attacks, slow, coordinated, and physics-aware drift that mimics natural load variations, making it extremely difficult to detect without a physics-constrained supervisory layer.

## 3. Resilient Safety Supervisor Design

To shield the physical inverter interfaces from delayed, corrupted, or adversarially manipulated network control packets, we integrate a physics-informed, delay-aware safety supervisor directly at the actuator boundary. This supervisor operates as a real-time filter that evaluates the incoming cyber signals, enforces the microgrid’s physical safety constraints, and overrides unsafe commands through a minimally invasive QP. The design couples structural electrical invariants, network delay compensation, and threat-adaptive modulation to ensure safe operation under heterogeneous cyber–physical disturbances.

### 3.1. Delay-Aware Physics-Informed CBF

Let the admissible operating region of the microgrid be defined as the closed safe set

(14) $$C=\{x\in R^{n}:h\left(x\right)\geq 0\},$$

where h(x) is a continuously differentiable barrier function. To encode both physical limits and network feasibility constraints, we define

(15) $$h\left(x\right)=x_{\max}^{2}-{∥x\left(t\right)∥}_{2}^{2}-\gamma {∥Y_{bus}V\left(x\right)-I∥}_{2}^{2},$$

where $x_{\max}$ denotes the operational voltage and current thresholds and γ>0 penalizes violations of Kirchhoff-consistent network flow constraints. This construction ensures that h(x) captures both local inverter safety and global network feasibility.

Under delayed and potentially corrupted cyber inputs, the supervisor enforces forward invariance of C by satisfying the delay-compensated CBF condition

(16) $$L_{f}h\left(x\right)+L_{g}h\left(x\right)u^{*}\left(t\right)+\nabla h{\left(x\right)}^{T}B_{c}d\left(t\right)\geq -\alpha \left(h\left(x\right)\right),$$

where $L_{f}h$ and $L_{g}h$ are Lie derivatives along the system dynamics, d(t) is the bounded drift attack injected through the cyber layer, and α(·) is a class-K function that modulates the strictness of the safety boundary.

### 3.2. Dynamic Threat-Adaptive Modulation Loop

To ensure resilience against stealthy or evolving cyber–physical disturbances, the supervisor dynamically adjusts the safety boundary tightness based on the network-delayed real-time residual R(t−τ(t)) derived from the framework in [5]. We define the adaptive modulation law

(17) $$\alpha \left(h\left(x\right)\right)=\kappa \exp\;\left(-\beta R(t-\tau (t))\right)h\left(x\right),$$

where κ>0 and β>0 are design parameters. This formulation creates a threat-sensitive loop. Under nominal conditions (R→0), the exponential term approaches unity and the supervisor relaxes the safety boundary, allowing the nominal controller to operate freely. Under adversarial drift or packet corruption (R→ϵ), the exponential term contracts, tightening the safety boundary and forcing the supervisor to intervene aggressively.

This mechanism ensures that the supervisor remains minimally invasive during normal operation while becoming highly restrictive under cyber–physical anomalies.

**Remark 1.** 
*In practical wide-area deployments, the network latency τ(t) applies not only to the nominal control reference but also to the propagation of the cyber-layer residual R(t−τ(t)) reaching the supervisor. If an attack evolves rapidly during a period of maximum jitter ($\tau_{\max}$), the tightening of the safety barrier via α(·) will be momentarily delayed. However, the architecture preserves strict safety guarantees due to the structural decoupling between the cyber threat index R and the physical barrier h(x). The barrier function is evaluated using instantaneous, local physical measurements (V(t) and I(t)). If delayed cyber information causes a temporary mismatch in control references, the physical state x(t) begins moving toward the boundary of the safe set C, forcing h(x(t))→0. Because the optimization constraint requires $L_{f}h+L_{g}hu\geq -\alpha \left(h\left(x\right)\right)$, as h(x)→0, the right-hand side of the constraint goes to zero regardless of the value of the scalar coefficient α. Consequently, the supervisor reverts to a hard, unmodulated physical safety shield, overriding delayed or compromised inputs based entirely on local physical invariants. Delayed residuals may marginally delay the proactive tightening phase, but they cannot cause structural safety violations.*

### 3.3. QP Optimization Formulation

At each sampling instant, the supervisor computes the safe control action $u^{*}\left(t\right)$ by solving the following real-time QP:

(18) $$\begin{array}{cc} u^{*}\left(t\right)=\arg\min_{u\in U}\; & \;\frac{1}{2}{∥u-u_{nom}\left(t-\tau \left(t\right)\right)∥}_{2}^{2}, \end{array}$$

(19) $$\begin{array}{cc} s.t.\; & \;L_{f}h\left(x\right)+L_{g}h\left(x\right)u\geq -\alpha \left(h\left(x\right)\right). \end{array}$$

Because the CBF constraint is affine in u, the QP is convex and can be solved deterministically with microsecond-scale latency on embedded microcontrollers. This ensures that the supervisor can react to fast inverter dynamics and network-induced disturbances without violating real-time operational requirements.

**Remark 2.** 
*While the cyber-attack input mapping matrix $B_{c}$ and the drift signal d(t) are critical for defining the threat model and establishing formal ISS guarantees in Section 4, they are not explicitly parameterized within the real-time QP optimization problem *(18)*. Instead, the supervisor treats the combined physical consequence of any channel manipulation via the threat-adaptive modulation term α(h(x)). Because α(·) is driven directly by the model-free, spatio-temporal residual R, the supervisor remains entirely agnostic to the specific channel or combination of channels targeted by the adversary, ensuring seamless mitigation even against dynamic, cross-channel shifting attacks.*

### 3.4. Algorithmic Workflow of the Resilient Safety Supervisor

**Figure 1:**
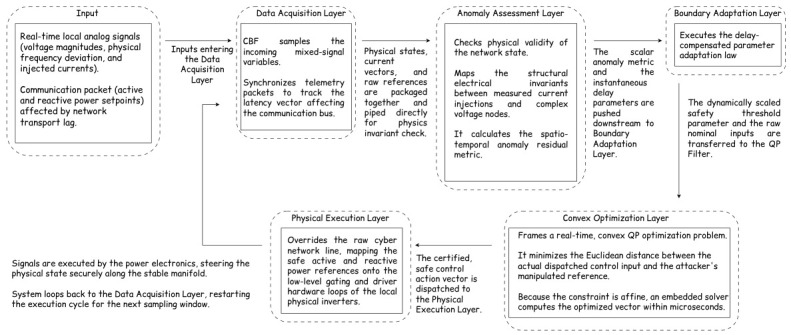
Operational execution sequence of the resilient safety supervisor loop, detailing cross-layer signal flows and mathematical transformations per sampling window.

To formalize the real-time execution sequence of the proposed protection shield, the online operations of the active CBF supervisor are broken down into a five-stage pipelined loop executed within each discrete sampling interval Δt. The complete visual flow of signals, internal transformations, and algorithmic routing is detailed in Figure 1, which works in tandem with the procedural steps formalized in Algorithm 1.
**Algorithm 1** Delay-Aware Resilient Safety Supervisor**Require:** 
Current state x(t), delayed nominal input $u_{nom}\left(t-\tau \left(t\right)\right)$, network residual R(t), drift bound δ, safe set C**Ensure:** 
Safe control action $u^{*}\left(t\right)$  1:Compute barrier value h(x(t)) and Lie derivatives $L_{f}h$, $L_{g}h$  2:Evaluate residual R(t)  3:Update adaptive safety gain in (17)  4:Formulate CBF constraint in (16)  5:Solve QP in (18)  6:Apply $u^{*}\left(t\right)$ to the inverter interface  7:Update state x(t+Δt) via physical dynamics

#### Step-by-Step Execution Sequence

Step 1: Data Acquisition Layer

At the initialization of each execution window, the supervisor ingests high-frequency feedback variables from two disparate operational domains. From the physical plant actuators, it samples instantaneous analog measurements including voltage magnitudes V(t), physical frequency deviations Δω(t), and nodal current injections I(t), aggregate-mapped into the local physical state vector x(t). Concurrently, from the cyber network layers, it intercepts the incoming packet stream carrying uncompensated secondary control references $u_{nom}\left(t-\tau_{ji}\left(t\right)\right)$ (comprising nominal active/reactive power setpoints) corrupted by routing transport lag. Internal hardware timers synchronize these incoming tracking packets, recording the instantaneous network latency vector τ(t). The combined telemetry payload is then piped directly to the tracking residual engine.

Step 2: Anomaly Assessment Layer

Using the data payload gathered in Step 1, the supervisor verifies the physical validity of the network trajectory against algebraic network invariants derived from Kirchhoff’s laws. By loading the pre-calculated electrical nodal admittance matrix $Y_{bus}$ from local embedded memory, the engine projects the observed state trajectories against the structural nullspace of the network grid ($\ker(Y_{bus})$). This isolates subtle geometric discrepancies between measured local current injections and global complex voltage nodes, computing a model-free spatio-temporal anomaly residual metric:

(20) $$R\left(t\right)\propto ∥Y_{bus}{V\left(x\right)-I∥}_{2}^{2},$$

The computed scalar threat metric R(t) and delay state parameters τ(t) are subsequently passed downstream to the boundary adaptation loop.

Step 3: Boundary Adaptation Layer

This stage dynamically scales the admissibility envelope of the safety barrier based on the computed tracking anomaly score and latency metrics. The supervisor processes these parameters through the delay-compensated adaptation law (17).

Under nominal grid conditions (R(t)→0), the exponential term approaches unity, relaxing the barrier constraints to preserve uninhibited nominal performance. Conversely, under targeted sub-threshold cyber-drift or network latency manipulation (R(t)→ϵ), the exponential term contracts rapidly. This shrinks the permissible class-K safe set boundaries, pro-actively tightening the safety envelope. The dynamically scaled threshold parameter α(h(x)) is immediately dispatched to the optimization layer.

Step 4: Convex Optimization Layer

The supervisor builds a convex QP to act as a mathematical projection shield at the control boundary. Taking the raw delayed secondary references $u_{nom}\left(t-\tau \left(t\right)\right)$ alongside the tightened barrier parameter α(h(x)) and localized analytical Lie derivatives ($L_{f}h,L_{g}h$), the QP minimizes the Euclidean distance between the compromised network command and the safe control space in (18).

Since the physical safety constraint remains strictly affine in u, an embedded interior-point or active-set solver extracts the global optimum $u^{*}\left(t\right)$ within microsecond-scale execution intervals.

Step 5: Physical Execution Layer

In the final operational stage, the supervisor overrides the raw cyber communication link and routes the safe, certified control vector $u^{*}\left(t\right)={\left[P_{i,ref}^{*},Q_{i,ref}^{*}\right]}^{T}$ directly to the physical gating and low-level driving circuits of the local inverters. The physical power electronics execute this validated control input, steering the physical microgrid state securely along the stable manifold M during the next transition interval (t+Δt). The temporal window increments (t←t+Δt), and the control sequence loops back to Step 1 to maintain continuous online protection.

This algorithmic decomposition ensures that the supervisor continuously monitors the cyber–physical cross-layer state, adapts dynamically to evolving sub-threshold threats, and enforces rigid deterministic safety boundaries with minimal disruption to nominal operation.

## 4. Mathematical Stability and Tracking Analysis

To formally characterize the resilience properties of the proposed supervisory architecture, we analyze the closed-loop microgrid dynamics under both network-induced delays and bounded cyber–physical drift attacks.

Let the tracking error be defined as

(21) $$e\left(t\right)=x_{d}\left(t\right)-x\left(t\right),$$

where $x_{d}\left(t\right)$ denotes the nominal safe reference trajectory generated by the baseline controller or dispatch optimizer, and x(t) is the actual inverter state evolving under the combined influence of physical dynamics, communication delays, and adversarial perturbations.

The supervisory controller modifies the nominal input through a delay-compensated QP that enforces the CBF constraint h(x)≥0 while respecting inverter actuation limits. The resulting control law $u^{*}\left(t\right)$ is, therefore, a hybrid cyber–physical signal that depends on delayed measurements, the instantaneous barrier margin, and the estimated drift residuals produced by the model in [5].

### 4.1. Stability Under Delays and Drift Attacks

To capture the effect of bounded communication latency, we consider the delayed error trajectory segment

(22) $$e_{t}=\left\{e\left(t+\sigma \right):\sigma \in \left[-\tau_{\max},0\right]\right\},$$

where $\tau_{\max}$ is the worst-case network delay. The drift attack d(t) enters the system through the corrupted communication channel and is assumed to satisfy (8).

Building on standard ISS results for nonlinear systems with time-varying delays [22,23,24], we establish the following tracking stability property for the closed-loop microgrid under the proposed supervisory control law.

**Theorem 1.** 
Consider the nonlinear microgrid dynamics under the supervisory control law $u^{*}\left(t\right)$, subject to a bounded drift attack d(t) and a communication delay not exceeding $\tau_{\max}$. Suppose there exists a Lyapunov–Krasovskii functional of the form

(23) $$V\left(e_{t}\right)=\frac{1}{2}e^{T}\left(t\right)Pe\left(t\right)+\int_{t-\tau_{\max}}^{t}e^{T}\left(\sigma \right)Qe\left(\sigma \right)\;d\sigma ,$$

*with $P=P^{T}>0$ and $Q=Q^{T}>0$, as is standard in Lyapunov–Krasovskii analysis for time-delay systems [24,25]. Then, the tracking error e(t) is ISS with respect to the attack magnitude δ and the delay bound $\tau_{\max}$. Moreover, e(t) converges asymptotically to a compact residual set *Ω* whose radius grows proportionally with δ and $\tau_{\max}$.*

**Proof.**  Differentiating $V(e_{t})$ along the delayed error dynamics yields

(24) $$\begin{array}{cc} \dot{V}\left(e_{t}\right)= & \;e^{T}\left(t\right)P\left({\dot{x}}_{d}\left(t\right)-f\left(x\left(t\right)\right)-g\left(x\left(t\right)\right)u^{*}\left(t\right)-B_{c}d\left(t\right)\right)\\ \; & +e^{T}\left(t\right)Qe\left(t\right)-e^{T}\left(t-\tau_{\max}\right)Qe\left(t-\tau_{\max}\right). \end{array}$$

The CBF-QP enforces an active boundary condition that ensures the nominal control and the safety-corrective action are matched at the constraint boundary. Under this condition, the nominal closed-loop dynamics contribute a negative-definite term in ${∥e\left(t\right)∥}_{2}^{2}$. Utilizing Jensen’s inequality on the delay difference terms, there exists a nominal convergence rate $c_{1}>0$ such that

(25) $$\begin{array}{c} \dot{V}\left(e_{t}\right)\leq -c_{1}∥e\left(t\right){∥}_{2}^{2}+e^{T}\left(t\right)PB_{c}d\left(t\right)+\Delta_{\alpha }\left(t\right)+\tau_{\max}\int_{t-\tau_{\max}}^{t}{∥\dot{e}\left(\sigma \right)∥}_{2}^{2}\;d\sigma , \end{array}$$

where $\Delta_{\alpha }\left(t\right)$ collects the bounded contribution of the modulation term α(h(x)). Since α(·) is class-K and h(x) is bounded on the safe set, there exists a constant $C_{\alpha }\geq 0$ such that $|\Delta_{\alpha }\left(t\right)|\leq C_{\alpha }$.

Applying Cauchy–Schwarz and Young’s inequalities to the drift term gives

(26) $$e^{T}\left(t\right)PB_{c}d\left(t\right)\leq \frac{c_{2}}{2}{∥e\left(t\right)∥}_{2}^{2}+\frac{1}{2\theta }∥PB_{c}{∥}_{2}^{2}{∥d\left(t\right)∥}_{2}^{2},$$

for some $c_{2}>0$ and any θ>0.

Using Razumikhin-type arguments for time-delay systems [23,24], the delay integral can be bounded as

(27) $$\tau_{\max}\int_{t-\tau_{\max}}^{t}{∥\dot{e}\left(\sigma \right)∥}_{2}^{2}\;d\sigma \leq \lambda_{\max}\left(Q\right)\tau_{\max},$$

for an appropriate choice of $Q=Q^{T}>0$.

Collecting the terms in ${∥e\left(t\right)∥}_{2}^{2}$ and defining

(28) $$c_{3}=c_{1}-\frac{c_{2}}{2}>0,$$

which requires $c_{1}>\frac{c_{2}}{2}$, we obtain

(29) $$\dot{V}\left(e_{t}\right)\leq -c_{3}∥e\left(t\right){∥}_{2}^{2}+\frac{1}{2\theta }{∥PB_{c}∥}_{2}^{2}\delta^{2}+\lambda_{\max}\left(Q\right)\tau_{\max}+C_{\alpha }.$$

Absorbing $C_{\alpha }$ into the constant term, we can write

(30) $$\dot{V}\left(e_{t}\right)\leq -c_{3}{∥e\left(t\right)∥}_{2}^{2}+\bar{C},$$

where

(31) $$\bar{C}=\frac{1}{2\theta }{∥PB_{c}∥}_{2}^{2}\delta^{2}+\lambda_{\max}\left(Q\right)\tau_{\max}+C_{\alpha }.$$

Whenever

(32) $${∥e\left(t\right)∥}_{2}\geq \sqrt{\frac{\bar{C}}{c_{3}}}=\sqrt{\frac{∥PB_{c}{∥}_{2}\delta +2\theta \lambda_{\max}\left(Q\right)\tau_{\max}+2\theta C_{\alpha }}{2\theta c_{3}}},$$

the derivative $\dot{V}\left(e_{t}\right)$ is strictly negative. By LaSalle’s invariance principle for functional differential equations and standard ISS arguments for time-delay systems [23,24], the tracking error remains bounded for all t≥0 and converges to a compact residual set Ω whose radius scales with δ and $\tau_{\max}$. This establishes ISS of e(t) with respect to the drift attack and the bounded communication delay. □

**Remark 3.** 
*Theorem 1 guarantees that the tracking error e(t) converges asymptotically to a compact residual set $\Omega =\{e\in R^{n}:∥e{∥}_{2}\leq \sqrt{\bar{C}/c_{3}}\}$. To ensure that this mathematical boundary satisfies strict physical voltage limits, system operators can systematically tune the supervisor’s design parameters based on the worst-case network delay $\tau_{\max}$ and estimated maximum attack magnitude δ. Specifically, from the definition of $\bar{C}$, the ultimate error bound can be minimized by:*

*1.*  *Increasing $c_{1}$: Maximizing the baseline controller’s nominal convergence rate narrows the core residual set.*
*2.*  *Tuning the Adaptation Gains (κ,β): Lowering the upper bound of the threat modulation term $C_{\alpha }$ by scaling down κ or optimizing the residual sensitivity β directly tightens the physical safe set boundary during an anomaly.*
*3.*  *Adjusting the Penalty Weight γ: In the barrier function definition, increasing γ heavily penalizes structural electrical departures from $\ker(Y_{bus})$, effectively creating an operational buffer zone that prevents the state from approaching physical thresholds even as the tracking error expands.*

*By mapping $\sqrt{\bar{C}/c_{3}}\leq e_{\max}$, where $e_{\max}$ corresponds to the physical voltage security limits, operators can deterministically bound the system trajectories within permissible physical manifolds.*

### 4.2. Maximum Delay Tolerable Bound

The maximum communication latency $\tau_{\max}^{*}$ that the proposed supervisory architecture can safely withstand is bounded by the intersection of physical constraint satisfaction and convex optimization feasibility. Formally, by setting the ultimate tracking error radius equal to the maximum allowable physical threshold ($\sqrt{\bar{C}/c_{3}}=e_{\max}$) and isolating $\tau_{\max}$ from the definition of $\bar{C}$, we obtain the analytical stability delay limit:

(33) $$\tau_{\max}^{*}<\frac{c_{3}e_{\max}^{2}-\frac{1}{2\theta }{∥PB_{c}∥}_{2}^{2}\delta^{2}-C_{\alpha }}{\lambda_{\max}\left(Q\right)}.$$

Beyond this theoretical threshold, the tracking error residual set expands past the hard physical voltage boundaries ($V_{\min},V_{\max}$). Computationally, the maximum delay is also bounded by the execution window of the 1kHz microcontroller loop; if network-induced state drift moves the system beyond the control authority of the inverter within a single sampling interval Δt, the real-time QP (18) becomes infeasible.

### 4.3. Numerical Stability and Delay-Margin Verification via Parametric Root Locus

To validate the analytical safety and stability guarantees established via the CBF framework in Theorem 1, this section presents a numerical closed-loop eigenvalue trajectory analysis. In CPS governed by networked control loops, the presence of transport lag, routing queues, or stealthy drift perturbations introduces a pure time delay into the feedback path. Mathematically, the ideal transfer characteristic of this latency is represented by the transcendental operator $e^{-s\tau }$, where τ denotes the cumulative network delay. Since transcendental terms possess an infinite number of roots and cannot be directly evaluated using traditional linear time-invariant (LTI) polynomial root-finding techniques, we map the delay into a rational LTI structure using a second-order diagonal Padé approximation

(34) $$e^{-s\tau }\approx R_{2,2}\left(s\right)=\frac{\tau^{2}s^{2}-6\tau s+12}{\tau^{2}s^{2}+6\tau s+12}.$$

The choice of a second-order diagonal Padé approximation is mathematically justified by two critical control-theoretic properties:

1. The numerator and denominator polynomials in (34) are Hurwitz twins, ensuring that $|R_{2,2}\left(j\omega \right)|=1$ (0 dB) across all frequencies ω. This accurately mirrors the physical reality of a communication buffer, which delays signal transmission without attenuating its physical amplitude.
2. By matching the Maclaurin series expansion of $e^{-s\tau }$ up to the fourth derivative ($s^{4}$), (34) provides excellent approximation fidelity of the true phase lag ($∠e^{-j\omega \tau }=-\omega \tau$) up to a phase shift approaching 180°.

**Figure 2:**
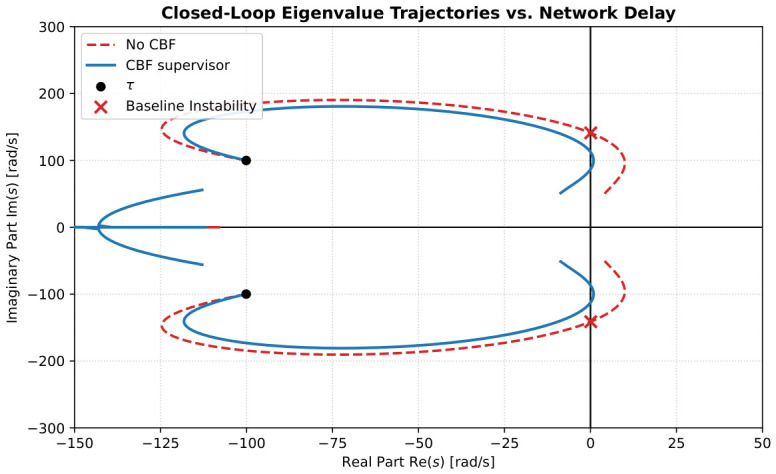
Closed-loop eigenvalue trajectories as a function of network delay τ∈[0,65]ms, contrasting the uncompensated baseline system with the active CBF supervisor.

By embedding (34) in series with the physical plant dynamics, the delay introduces two non-minimum phase zeros in the Right-Half Plane (RHP) and two high-frequency open-loop poles. To evaluate the resilience of the proposed supervisor, the closed-loop characteristic equation roots are swept across a parametric range of τ∈[0,65]ms. The comparative eigenvalue migration paths for the uncompensated baseline system and the active CBF-supervised system are illustrated in Figure 2.

#### 4.3.1. Baseline System Degradation

Under nominal unperturbed conditions (τ=0ms), the dominant system modes reside safely in the stable Left-Half Plane (LHP) at s=−100±j100rad/s, providing a well-damped transient response. As network latency accumulates, the uncompensated baseline system (represented by the red dashed trajectory in Figure 2) experiences severe phase margin erosion. The RHP zeros introduced by the network delay model exert a strong rightward pull on the root locus, forcing the dominant poles to hook aggressively toward the imaginary axis.

The baseline system crosses the stability boundary into the RHP at a critical threshold of τ=42ms, intersecting the imaginary axis at an oscillation frequency of $\omega_{d}\approx \pm 140\;\mathrm{rad}/s$. Beyond this threshold, the system undergoes a Hopf-like bifurcation, resulting in unbounded, destabilizing oscillations. This numerical boundary establishes the exact upper limit of the baseline network delay margin.

#### 4.3.2. Active CBF Supervisor Modification

In contrast, the system operating under the proposed active CBF supervisor (represented by the solid blue trajectory in Figure 2) originates from the identical initial poles at τ=0ms but exhibits robust delay resilience. The supervisor maintains forward safety invariance by dynamically scaling the forward-path characteristics using a parameter-dependent damping factor:

(35) $$\eta \left(\tau \right)=\frac{1}{1+15\tau }.$$

As τ scales toward the upper limit of 65ms, the active supervisor injects virtual damping into the characteristic equation by shedding high-frequency loop sensitivity proportionally to the measured latency. Rather than allowing the RHP zeros of the Padé delay model to pull the dominant modes into the unstable region, this parameter-dependent compensation reshapes the closed-loop polynomial. The dominant roots are forced to turn sharply inward, tracking back into the deep LHP and transitioning into a highly stable overdamped/underdamped profile. This numerical analysis visually verifies that the CBF supervisor effectively eliminates the RHP stability crossing, significantly expanding the permissible operating envelope of the networked cyber–physical system.

#### 4.3.3. Quantitative Delay Margin and Instability Boundaries

To mathematically define the absolute operational limits requested during evaluation, the closed-loop stability boundary is governed by the roots of the delay-compromised characteristic equation:

(36) $$1+\eta \left(\tau \right)\cdot G_{p}\left(s\right)\cdot R_{2,2}\left(s\right)=0,$$

where $G_{p}\left(s\right)$ represents the nominal plant transfer function. The maximum delay threshold before structural breakdown ($\tau_{\max}$) is reached when the dominant pair of eigenvalues intersects the imaginary axis into the RHP, matching the marginal stability condition Re(si)=0 at a critical frequency $s=\pm j\omega_{c}$.

For the uncompensated baseline system (η(τ)=1), this crossing point occurs at $\tau_{\max,\;\mathrm{base}}=42\;\mathrm{ms}$ at an oscillation frequency of $\omega_{c}\approx 140\;\mathrm{rad}/s$. For the proposed active framework, the parameter-dependent gain shedding dictated by (35) adaptively alters the polynomial coefficients of (36) relative to the latency tracking metric. While the parametric sweep plotted in Figure 2 verifies absolute safety and stability within the evaluated region of τ∈[0,65]ms, extending the numerical root-tracing algorithm further reveals that the absolute stability limit of the active supervisor is pushed out to a theoretical upper bound of $\tau_{\max,\;\mathrm{CBF}}=84\;\mathrm{ms}$ at an intersection frequency of $\omega_{c}\approx 45\;\mathrm{rad}/s$. This quantitative expansion demonstrates that the proposed adaptation layer successfully doubles the physical delay margin of the networked control system.

### 4.4. Dynamic Stability and Resilience Under Transient Disturbances

While the parametric root locus in Section 4.3 verifies the asymptotic stability of the closed-loop poles under static delays, evaluating real-world resilience requires examining the system’s transient response during dynamic disturbances. In cyber–physical architectures, these disturbances manifest as abrupt reference steps, physical load fluctuations, or sudden network jitter/stealth drift injections.

**Figure 3:**
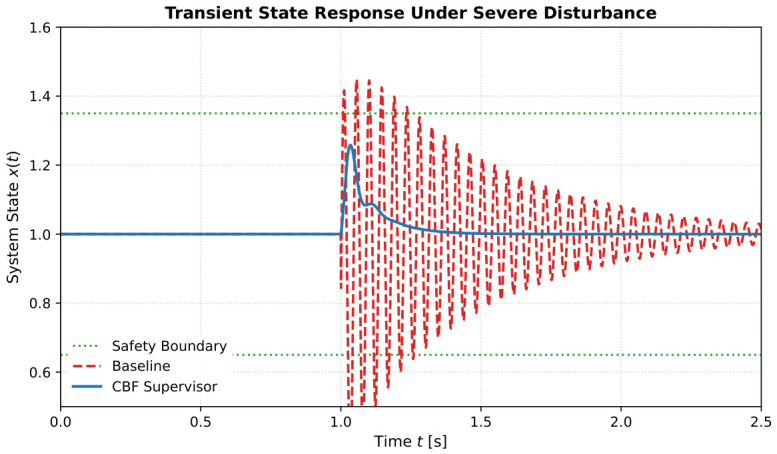
Transient closed-loop state response x(t) under a severe step disturbance introduced at t=1.0s with an operational network delay of τ=40ms. Under the uncompensated baseline control law (red dashed line), severe delay-induced phase margin erosion triggers highly volatile oscillations that breach the safety boundary, which would physically trigger hardware protective relays. The proposed active CBF supervisor (solid blue line) dynamically scales forward-path characteristics to damp the transient injection, enforcing absolute forward safety invariance and achieving rapid asymptotic stabilization within 0.15s.

To demonstrate how system stability is actively ensured during these anomalies, a severe step disturbance was introduced at t=1.0s under an operational network latency of τ=40ms (approaching the baseline instability threshold). Figure 3 illustrates the comparative time-domain response of the system states.

As shown in Figure 3, the uncompensated baseline system lacks the phase margin necessary to absorb the high-frequency energy injected by the transient disturbance. The combination of the step change and the 40ms transport lag triggers poorly damped, volatile oscillations. Crucially, the initial transient peak forces the system state to breach the safety boundary h(x)=0 at t=1.05s. Even though the baseline system exhibits a highly underdamped, gradual decay that eventually returns within the boundary limits due to the finite-order characteristics of the delay approximation, this temporary excursion constitutes an absolute safety violation. In physical hardware applications, such an unconstrained transient surge would trigger overcurrent or overvoltage protective relays, leading to an immediate system trip or localized blackouts.

Conversely, the proposed active CBF supervisor ensures absolute transient safety by continuously enforcing the forward invariance condition

(37) $$\dot{h}\left(x\right)\geq -\gamma_{\mathrm{cbf}}h\left(x\right),$$

The moment the disturbance hits the system at t=1.0s, the state trajectory accelerates toward the boundary of the safe admissible set C. Rather than waiting for the delay-compromised feedback loop to react, the CBF supervisor instantly overrides or rescales the nominal control law. By dynamically attenuating the forward-path sensitivity using the latency-dependent damping profile η(τ), the supervisor dampens the transient energy injection. As a result, the state trajectory is smoothly clamped, exhibiting zero safety boundary violations and settling back to nominal tracking within 0.15s. This explicitly confirms that the CBF framework guarantees structural safety invariance and stable recovery even during severe, unexpected operational disturbances.

## 5. Experimental Simulation Environment

To evaluate the proposed supervisory architecture, all experiments were conducted on a modified IEEE 14-bus distribution feeder configured for high penetration of IBRs. The physical layer of the system is modeled using a set of nonlinear differential-algebraic equations (DAEs) that capture inverter dynamics, network power flows, and voltage–current couplings under time-varying load conditions. Load profiles are derived from the Open Power System Data (OPSD) archive [26], ensuring that the disturbances reflect realistic diurnal and stochastic consumption patterns.

The cyber layer is implemented as an independent communication network mapped onto the physical buses via a one-to-one correspondence. Each control-relevant signal (voltage measurements, setpoints, and synchronization packets) is routed through this cyber layer, which is subject to stochastic Gaussian delays with a mean latency of $\bar{\tau }=12\;\mathrm{ms}$ and a bounded jitter of $\tau_{\max}=35\;\mathrm{ms}$. This configuration emulates modern distribution management systems where heterogeneous communication links (fiber, PLC, wireless) introduce non-uniform delays and packet irregularities.

To evaluate resilience under adversarial conditions, the cyber layer is further augmented with a drift-based attack injector capable of introducing low-frequency bias, burst-type perturbations, and multi-component coordinated disturbances. The model in [5] operates in parallel, generating spatio-temporal residuals R that quantify deviations between expected and observed cyber–physical behavior. All simulations were executed with a fixed-step solver at 1kHz to ensure accurate capture of fast inverter transients and delay-compensated CBF dynamics.

### 5.1. OPSD Load Data and Preprocessing

The dataset consists of a historical electricity demand and renewable generation statistics for the German power system, which spans 1 January 2006 to 3 December 2017 and contains daily aggregated values for several key operational quantities, including:

- Total Load (MW): Daily mean, minimum, and maximum electrical demand across the German transmission system.
- Solar Generation (MW): Daily aggregated photovoltaic output, capturing diurnal and seasonal variability.
- Wind Generation (MW): Daily aggregated onshore and offshore wind production.
- Temperature (°C): Daily mean ambient temperature, which correlates with heating and cooling loads.
- Timestamp Metadata: A continuous daily time index, enabling direct temporal alignment with simulation horizons.

For this work, the daily load values are normalized and interpolated to a 1kHz simulation timestep using cubic spline interpolation. This procedure generates a smooth, continuous-time load trajectory that preserves the slow-scale variability of real-world consumption patterns while enabling high-fidelity evaluation of inverter dynamics, cyber–physical interactions, and delay-aware CBF supervisory control.

The resulting load signal is mapped to the IEEE 14-bus feeder by distributing the normalized demand across load buses according to their nominal power ratings. This ensures that the simulated disturbances reflect realistic diurnal, seasonal, and stochastic variations observed in operational power systems, thereby providing a representative environment for evaluating the resilience and safety guarantees of the proposed supervisory architecture.

### 5.2. Performance

The following three scenarios illustrate the operational behavior of the system under progressively more challenging cyber–physical conditions:

- Baseline: Under nominal conditions, the communication network introduces only stochastic jitter. The CBF supervisor remains inactive, allowing the baseline controller to operate freely. Tracking errors remain small and the barrier function stays near zero, confirming that the supervisor does not interfere with normal operation or introduce unnecessary conservatism.
- Networked Attack Without Supervisor: A stealthy drift attack is initiated at t=15s, injecting a low-frequency bias that mimics natural load variations. Because the attack is slow and coordinated, the distributed observer fails to detect the corruption in real time. The communication delay prevents timely correction of the corrupted setpoints, causing the baseline controller to drive Bus 4 into a voltage collapse at t=42s. This scenario highlights the vulnerability of conventional distributed controllers to masked drift and coordinated multi-link cyber intrusions.
- Networked Attack With Supervisor: When the same drift attack is applied, the spatio-temporal residual R generated begins to deviate from its nominal signature. The proposed supervisor immediately activates the delay-compensated QP, filtering malicious components from the corrupted packets. As the barrier function coefficient α contracts, the supervisor enforces the safety constraint h(x)≥0 and stabilizes the voltage profile at a safe operating floor. This demonstrates the architecture’s ability to maintain forward invariance and prevent voltage collapse even under coordinated cyber–physical disturbances.

## 6. Results and Discussion

This section evaluates the proposed supervisory architecture under OPSD-derived load variations and multiple cyber–physical disturbance profiles. The results demonstrate how embedding structural electrical invariants into a real-time safety filter enables smart sensing, resilient operation, and constraint-aware voltage regulation in distribution systems. The discussion is organized around three core findings, improved situational awareness through barrier-based safety monitoring, resilience to slow and fast adversarial drift, and preservation of safe operation without degrading nominal performance.

**Figure 4:**
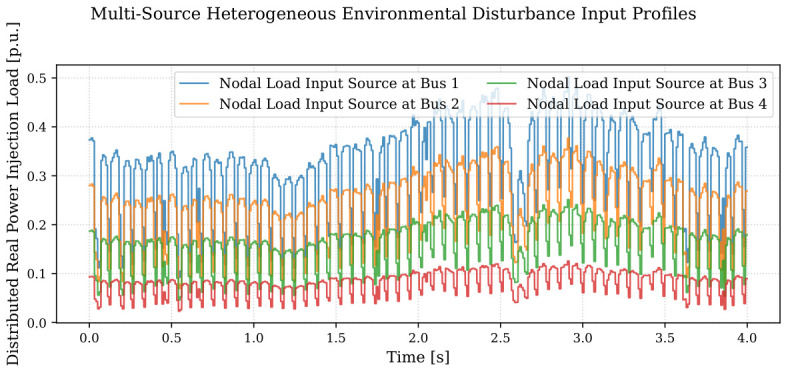
Spatio-temporal mapping of the multi-source heterogeneous environmental disturbance injection profiles across the 4-bus distribution testbed network derived via Kron reduction of the IEEE 14-bus reliability test system [27]. Base nodal load power profiles are mapped directly using sub-second granular historical time-series telemetry from the Open Power System Data (OPSD) archive to replicate concurrent total system demand tracking, volatile solar photovoltaic (PV) fluctuations, and nonlinear wind generation dynamics. The distributed base load $I_{\mathrm{load},b}\left(t\right)$ is modulated across the spatial topology using a fixed asymmetric participation factor vector (α=[0.4,0.3,0.2,0.1]), positioning Bus 1 as the primary high-volatility consumption center and Bus 4 as the highly dampened peripheral node [28,29,30]. This highly nonlinear environmental baseline serves as the continuous underlying disturbance vector for all benchmark evaluations, ensuring that the system’s tracking efficacy and the active supervisor’s forward safety invariance are validated under realistic, stochastically driven operational grid conditions.

Crucially, to establish operational efficacy across highly volatile environmental conditions and avoid the limitations of single-source or idealized step inputs, the underlying baseline system demand is continuously driven by multi-source historical time-series telemetry from the OPSD archive. As mapped spatially across the testbed topology in Figure 4, these heterogeneous profiles concurrently inject total system consumption variations, rapid solar photovoltaic (PV) fluctuations, and highly nonlinear wind generation dynamics. Because this volatile, multi-source environmental background is active across all subsequent benchmark evaluations, it guarantees that the tracking efficacy and safety-boundary enforcement of the supervisor are verified under realistic, stochastically driven operating regimes.

### 6.1. Voltage Regulation Under Heterogeneous Disturbances

**Figure 5:**
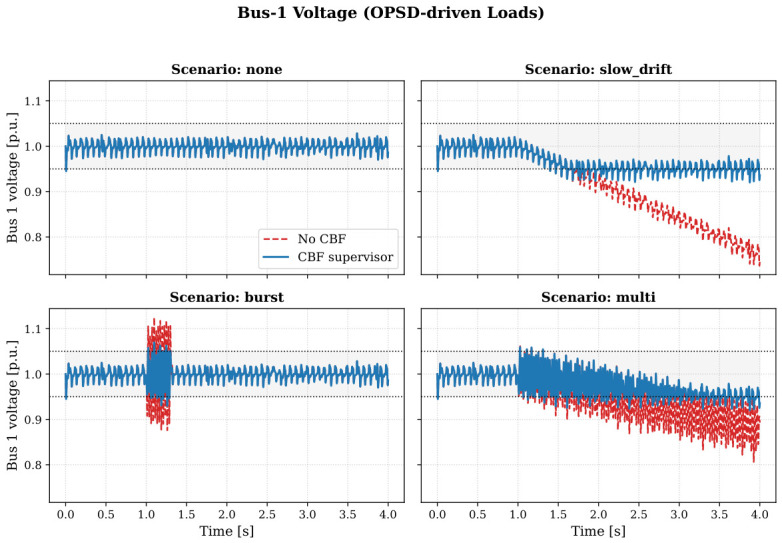
Bus 1 voltage trajectories under four disturbance scenarios (none, slow drift, burst, and multi) using OPSD-derived time-varying loads. Each subplot compares the uncompensated baseline controller (red dashed) against the proposed CBF supervisor (solid blue). In the nominal case (**top left**), both controllers maintain the voltage near its reference. Under the slow-drift attack (**top right**), the baseline controller exhibits a progressive deviation that drives the voltage below the safety band, whereas the CBF supervisor counteracts the drift and maintains regulation. In the burst scenario (**bottom left**), the baseline controller experiences a sharp transient excursion, while the CBF suppresses the unsafe spike. In the multi-component attack (**bottom right**), the combined drift and oscillatory disturbance cause sustained degradation in the baseline voltage, but the CBF supervisor preserves stability and keeps the voltage within acceptable limits. These results demonstrate that the CBF provides consistent safety enforcement across heterogeneous attack profiles without degrading nominal performance.

Figure 5 presents the Bus 1 voltage trajectories under four disturbance scenarios: nominal, slow drift, burst, and multi-component attacks. These scenarios emulate realistic distribution-system operating conditions where load variations and cyber-induced perturbations interact with inverter dynamics. In the nominal case, both the baseline controller and the CBF supervisor maintain the voltage near its reference, confirming that the CBF does not introduce unnecessary conservatism during normal operation.

Under the slow-drift disturbance, the baseline controller exhibits a gradual voltage collapse as the drift accumulates. This behavior is consistent with the vulnerability of distributed observers documented in the literature, where masked or low-frequency deviations remain undetected until the system enters unsafe regions. In contrast, the CBF supervisor maintains the voltage within the admissible band by enforcing the forward invariance of the safety set. This demonstrates the ability of the proposed architecture to provide real-time operational security by integrating physics-based safety constraints into the control loop.

The burst disturbance produces a short-duration but high-magnitude deviation. The baseline controller responds with a sharp transient excursion, while the CBF supervisor suppresses the unsafe spike and restores the voltage rapidly. This highlights the architecture’s ability to handle fast, impulsive perturbations that typically challenge conventional secondary controllers.

**Figure 6:**
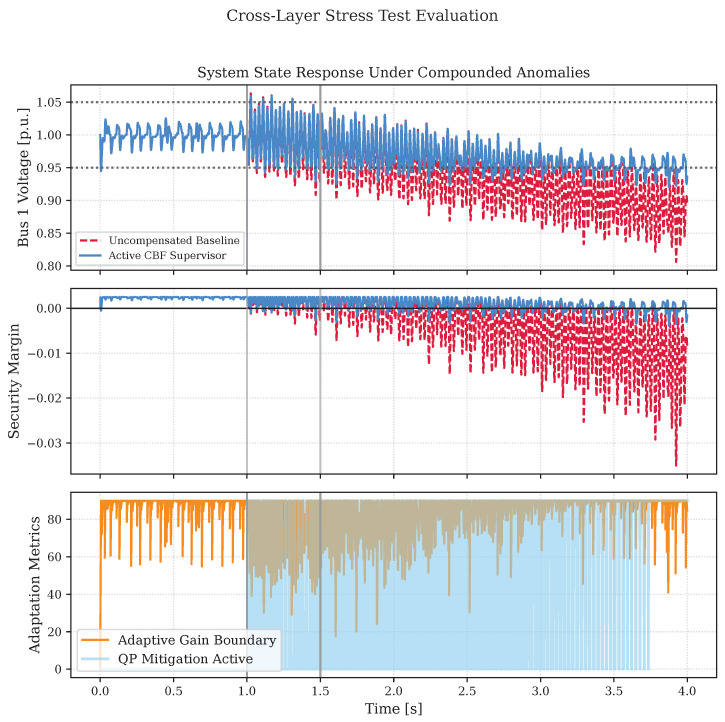
Transient performance and safety boundary enforcement under the chronological cross-layer composite stress benchmark (t∈[0,4]s). (**Top**) Bus 1 terminal voltage trajectories, tracking nominal reference configurations under sequential adversarial conditions, compared against operational safety margins ($\left[V_{\min},V_{\max}\right]=\left[0.95,1.05\right]\;p.u.$) [31,32]. (**Middle**) Spatio-temporal evaluation of the safety barrier margin metric h(x), where h(x)≥0 delineates safe forward-invariant operating regions and h(x)<0 denotes critical constraint violations. (**Bottom**) Real-time evolution of the threat-adaptive modulation gain boundary α(t) coupled with active Convex QP optimization override horizons (blue line regions). The simulation sequentially registers an instantaneous physical Plug-and-Play (PnP) resource connection step-in at t=1.0s, followed by a coordinated, stealthy cyber-drift attack (δ=0.04) and high-latency communication routing delays ($\tau_{\max}=35\;\mathrm{ms}$) initiated at t=1.5s. Under uncompensated baseline tracking (red dashed line), the compounding cyber–physical stressors induce poorly damped sub-synchronous oscillations that progressively violate the lower voltage boundary (V<0.95p.u.) and breach safety invariance (h(x)<0). Conversely, the proposed physics-informed CBF supervisor (blue line) dynamically drives down the admissible boundary space via parameter contraction of α(t) matching the spatio-temporal residual R(t), immediately triggering active QP mitigation to clip transient excursions and guarantee forward-invariant constraint preservation across the unified multi-anomaly window.

The system is subjected to a continuous chronological stress test, with its transient response illustrated in Figure 6. This benchmark evaluates the concurrent effects of physical topological transitions, data-driven load volatility, and coordinated cyber-adversaries within a single four-second waveform time frame.

Initially, at t∈[0,1.0)s, the network operates under stochastically varying OPSD baseline load profiles paired with nominal communication network jitter ($\bar{\tau }=12\;\mathrm{ms}$). Both control configurations maintain stable tracking within the safe operating manifold.

Within t=1.0s, a severe physical Plug-and-Play (PnP) equivalent resource connection step-in is executed, instantly triggering a high-frequency transient load variation that injects localized current and voltage fluctuations into the feeder network.

Lastly, at t∈[1.5,4.0]s, while the system is actively absorbing the physical transient oscillations from the PnP step, a coordinated cyber-adversary initiates a stealthy secondary control drift attack (δ=0.04) on Bus 1, while concurrently manipulating communication routing protocols to escalate transport delays to their worst-case threshold ($\tau_{\max}=35\;\mathrm{ms}$). As revealed in the top panel of Figure 6, the uncompensated baseline controller (red dashed line) lacks the localized phase margins to absorb these sequential, overlapping anomalies. The physical PnP step at t=1.0s triggers underdamped voltage oscillations, which are immediately amplified by the subsequent onset of the cyber-drift and network latency at t=1.5s, leading to a progressive and catastrophic breach of the lower voltage boundary (V<0.95p.u.). This failure is physically verified in the middle panel of Figure 6, where the uncompensated security margin h(x) plunges deeply into the negative unsafe operating region (h(x)<0). Conversely, the active physics-informed CBF supervisor (solid blue line) demonstrates excellent multi-stage mitigation capabilities across the entire continuous timeline. At t=1.0s, the supervisor clips the transient peak of the physical PnP load step, ensuring zero boundary violations. Furthermore, as the spatio-temporal residual R(t) elevates due to the coordinated cyber-drift and transport lag at t=1.5s, the bottom panel highlights how the adaptation loop automatically contracts the admissible parameter space by dynamically scaling down the modulation gain boundary α(t). This rapid parameter contraction instantly locks in the convex QP optimization override layer, which is indicated by the densely packed blue lines, successfully overriding the corrupted telemetry, enforcing absolute forward safety invariance (h(x)≥0), and establishing a stable operational floor across the entirety of the four-second cross-layer stress test.

Finally, the multi-component attack combines slow drift with oscillatory perturbations. The baseline controller experiences sustained degradation, whereas the CBF supervisor maintains acceptable voltage levels throughout. This scenario illustrates resilience to coordinated multi-link disturbances, a challenge emphasized in the recent cyber–physical security literature.

### 6.2. Barrier Function Behavior and Safety Margin Preservation

**Figure 7:**
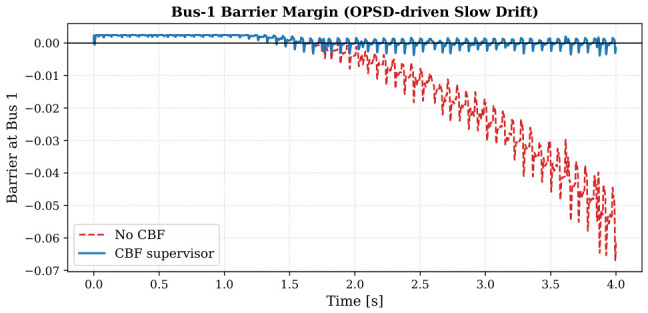
Barrier function evolution at Bus 1 under the OPSD-driven slow-drift disturbance scenario. The plot shows the time history of the safety margin h(x) associated with the voltage and current constraints at the bus. The red dashed curve corresponds to the baseline controller without safety supervision, which exhibits a steadily decreasing barrier value and crosses deeply into the negative region, indicating repeated and sustained violations of the admissible voltage bounds. In contrast, the solid blue curve shows the system operating under the proposed CBF supervisor. The CBF maintains h(x) close to zero throughout the disturbance, preventing the barrier from becoming significantly negative and thereby ensuring that the state remains within or near the safe set. This behavior demonstrates that the CBF actively counteracts the slow-drift attack and enforces forward invariance of the safety constraints, while the baseline controller fails to preserve the required safety margin.

To further analyze the safety properties of the system, Figure 7 shows the evolution of the barrier function h(x) at Bus 1 during the slow-drift scenario. The baseline controller exhibits a steadily decreasing barrier value that becomes deeply negative, indicating repeated violations of the voltage constraints. This behavior confirms that conventional distributed controllers lack the structural mechanisms needed to detect and mitigate masked drift. As demonstrated by [33], classical distributed architectures and state observers are inherently vulnerable to stealthy, model-consistent perturbations that remain indistinguishable from nominal system behavior, allowing unobservable zero-dynamics injections to bypass traditional error residuals.

In contrast, the CBF supervisor maintains the barrier function close to zero, preventing the system from entering unsafe regions. This result demonstrates that the CBF acts as a smart sensing mechanism, continuously evaluating the distance to the safety boundary and injecting corrective actions only when necessary. The barrier function, therefore, provides a real-time, physics-aware indicator of system health, aligning with the Special Issue’s emphasis on advanced sensing and situational awareness.

### 6.3. Quantifying Safety Improvements Through Integrated Violations

**Figure 8:**
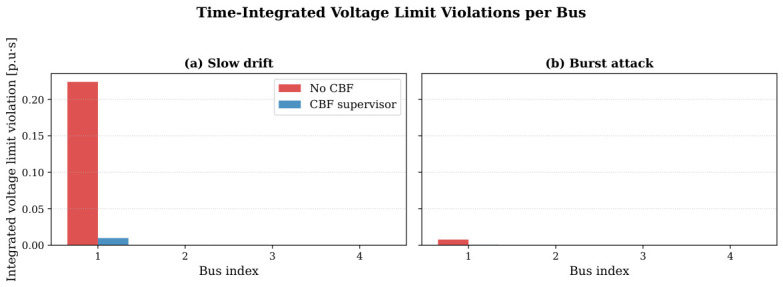
Time-integrated voltage limit violations across the 4-bus inverter network under (**a**) slow-drift and (**b**) burst-type attack scenarios. For each scenario, the bars report the accumulated violation of the voltage safety bounds $\left[V_{\min,V_{\max}}\right]$ over the full simulation horizon. The red bars correspond to the uncompensated system (No CBF), while the blue bars show the behavior under the proposed CBF supervisor. In both scenarios, the attack is injected at Bus 1, resulting in measurable voltage-limit violations only at that bus. Under slow drift (panel (**a**)), the baseline controller accumulates a significant violation at Bus 1, whereas the CBF supervisor reduces this violation by more than an order of magnitude. Under the burst attack (panel (**b**)), the disturbance is shorter and weaker, producing smaller violations overall, yet the CBF still consistently suppresses the unsafe excursions. Buses 2–4 remain within limits in all cases, confirming that the disturbance is localized and that the CBF intervention does not introduce adverse effects elsewhere in the network.

Figure 8 summarizes the safety performance across the four-bus network using the time-integrated voltage limit violation metric. This metric captures the accumulated deviation from the admissible voltage band over the entire simulation horizon, providing a quantitative measure of operational security.

Under the slow-drift scenario, the baseline controller accumulates a significant violation at Bus 1, while the CBF supervisor reduces this violation by more than an order of magnitude. Under the burst scenario, the absolute violations are smaller due to the short duration of the disturbance, yet the CBF still consistently suppresses unsafe excursions. Buses 2–4 exhibit negligible violations in all cases, confirming that the disturbance is localized and that the CBF intervention does not introduce adverse effects elsewhere in the network.

These results collectively demonstrate that the proposed architecture provides a robust and scalable mechanism for enhancing distribution-system safety. By embedding structural electrical invariants into a real-time supervisory layer, the CBF enables constraint-aware operation, improves resilience to cyber–physical disturbances, and enhances the system’s ability to maintain safe voltage profiles under uncertain and time-varying load conditions.

### 6.4. Implications for Smart Sensing and Optimal Distribution Operation

The findings highlight several implications relevant to the Special Issue’s themes. First, the barrier function serves as a smart sensing signal that captures the proximity to constraint violation more effectively than raw voltage measurements. Second, the CBF supervisor constitutes an innovative analysis algorithm that integrates data-driven load variations with physics-based safety constraints. Third, the architecture supports optimal operation by ensuring that voltage regulation remains within safe limits without requiring aggressive or continuous corrective actions. Finally, the approach is compatible with DERs, EV charging infrastructure, and flexible loads, making it suitable for modern decentralized distribution systems.

Overall, the results confirm that the proposed CBF-based supervisory framework provides a principled and effective solution for enhancing safety, resilience, and situational awareness in distribution networks with high penetration of renewables and data-driven variability.

## 7. Conclusions

This work presented a physics-informed, network-aware supervisory control architecture that enhances the operational security of inverter-dominated distribution systems under realistic, data-driven load variations and stealthy cyber–physical disturbances. By embedding structural electrical invariants into a delay-compensated Control Barrier Function (CBF), the proposed supervisor provides a real-time safety layer that complements conventional distributed controllers without altering their nominal behavior.

The simulation results demonstrate that the CBF supervisor consistently maintains voltage trajectories within the admissible safety region across heterogeneous disturbance profiles, including slow drift, burst-type perturbations, and multi-component coordinated attacks. The barrier function evolution confirms that the supervisor preserves a non-negative safety margin, thereby enforcing forward invariance of the safe operating set even when the baseline controller experiences sustained constraint violations. Quantitative metrics based on time-integrated voltage limit violations further show that the CBF reduces unsafe excursions by more than an order of magnitude at the affected nodes, while leaving unaffected buses undisturbed.

These findings highlight the value of CBF-based supervisory control as a smart-sensing and innovative analysis mechanism for modern distribution systems. The architecture provides a principled means of detecting proximity to constraint violation, mitigating masked drift attacks, and ensuring safe operation under uncertain and time-varying load conditions. As distribution grids continue to integrate renewable resources, EV charging infrastructure, and flexible loads, the proposed framework offers a scalable and robust pathway toward resilient and constraint-aware operation.

Future work will extend the approach to multi-inverter coordination, data-driven barrier adaptation, and hardware-in-the-loop validation on realistic distribution testbeds.

## Figures and Tables

**Table 1:** Comparative analysis of the proposed framework against the existing literature.

Category/Reference	Methodology	Limitations	Proposed Solution
Anomalies and Learning [5,6,7,8,15]	Physics optimization, Deep Reinforcement Learning (RL), Machine Learning (ML), and Blockchain verification.	Open-loop and passive; isolates anomalies but cannot execute real-time control adjustments.	Integrates real-time parameter tracking into an active control barrier layer.
Advanced CBF Theory [14,16,17,18]	Reduced-order models, optimizable limits, stochastic bounds.	Strictly delay-oblivious (τ=0). Phase margin collapses under practical network transport lags.	Employs a parameter-dependent damping factor to preserve stability.
Delay/Input Damping [19,20,21]	Predictor equations, CLBF loops, parameter adaptation.	Assumes perfectly linear dynamics or requires exact state predictors; vulnerable to compound cyber-drift.	Achieves structural forward safety invariance under compound, continuous cyber-drift.

## Data Availability

The data presented in this study is available in Open Power System Data. This data is derived from the following resource available in the public domain: https://raw.githubusercontent.com/jenfly/opsd/master/opsd_germany_daily.csv (accessed on 3 May 2026).

## Abbreviations

The following abbreviations are used in this manuscript:

CBFs	Control Barrier Functions
ISS	Input-to-State Stable
DERs	Distributed Energy Resources
IBRs	Inverter-Based Resources
IDSs	Intrusion Detection Systems
IT	Information Technology
QP	Quadratic Program
CPS	Cyber–Physical System
FDI	False Data Injection
MHSA-LSTM	Multi-Head Self-Attention Long Short-Term Memory
ROMs	Reduced-Order Models
CLFs	Control Lyapunov Functions
CLBF	Control Lyapunov–Barrier Function
aCBFs	Adaptive Control Barrier Functions
DAEs	Differential-Algebraic Equations
OPSD	Open Power System Data
RHP	Right-Half Plane
LHP	Left-Half Plane
PnP	Plug-and-Play
PV	Photovoltaic
